# Gr-1+CD11b+ myeloid cells efficiently home to site of injury after intravenous administration and enhance diabetic wound healing by neoangiogenesis

**DOI:** 10.1111/jcmm.12265

**Published:** 2014-03-19

**Authors:** Xiaozhe Tong, Gang Lv, Jianhua Huang, Yongfen Min, Li Yang, Pengnian Charles Lin

**Affiliations:** a Key Laboratory of Medical Tissue Engineering of Liaoning Province, First Affiliated Hospital of Liaoning Medical UniversityJinzhou, Liaoning, China; b Department of Traditional Chinese Medicine, First Affiliated Hospital of Liaoning Medical UniversityJinzhou, Liaoning, China; c Department of Radiation Oncology, Vanderbilt University School of MedicineNashville, TN, USA; d Department of Cardiac Surgery, Ningxia People's HospitalYinchuan, Ningxia, China; e Center for Cancer Research, National Cancer Institute-FrederickFrederick, MD, USA

**Keywords:** Gr-1+CD11b+ myeloid cells, homing, neoangiogenesis, diabetic wound healing

## Abstract

Vascularization is an important factor that affects diabetic wound healing. There is increasing evidence that myeloid cell lineages play a role in neovascularization. In this study, the efficiency of Gr-1+CD11b+ myeloid cells to home to the site of injury and enhance diabetic wound healing by neoangiogenesis after intravenous administration was investigated. Gr-1+CD11b+ myeloid cells were injected into tail vein after establishment of dorsal window chamber, hindlimb ischaemia and ear-punch injury in diabetic or non-diabetic mice. The Gr-1+CD11b+ myeloid cells efficiently homed to the site of injury after intravenous administration and increased neoangiogenesis. The chemokine receptor type 4 (CXCR4) is robustly expressed by Gr-1+CD11b+ myeloid cells. Inhibition of CXCR4 decreases the homing ability of Gr-1+CD11b+ myeloid cells to the site of injury, which indicates that the CXCR4/SDF-1 axis plays an important role in the homing of Gr-1+CD11b+ myeloid cells to the site of injury. In addition, Gr-1+CD11b+ myeloid cells were found to improve blood flow recovery of ischaemic limb and enhance wound healing in diabetic mice by neoangiogenesis after intravenous administration. Taken together, the results of this study suggest that Gr-1+CD11b+ myeloid cells may serve as a potential cell therapy for diabetic wound healing.

## Introduction

Skin is the first organ to protect the human from microorganisms, physical and chemical damage in the living environment. When skin damage occurs, the skin undergoes a series of biological and molecular events of repair including haemostasis, inflammation, proliferation, production, and remodelling of matrix and vascularization [1]. However, in diabetic patients, the wound healing is often impaired [2]. A central pathological factor in impaired wound healing is insufficient blood supply [3,4]. Although traditional physical, pharmacological and surgical methods are used to deal with wound repair in diabetic patients, the results are presently unsatisfactory. It is estimated that diabetic foot ulceration occurs in 15% of diabetic patients, causing prolonged hospitalization and even amputation [5]. Recently, cell therapy has emerged as an alternative and promising method for many diseases including the wound repair. For example, mesenchymal stem cells (MSCs) have been reported to accelerate diabetic wound healing *via* increasing angiogenesis both in animal models [4,6,7] and in a clinical trial [8]. However, a significant obstacle in clinical stem cell therapy is the low efficiency of homing and engraftment of stem cells to the site of injury after routine systemic or local administration. Thus, a stem cell population that has high efficiency of homing and engrafting to the site of injury is urgently required in clinical practice.

Among various sources of stem cell, the myeloid lineage cells have been demonstrated to play an essential role in adult neoangiogenesis [9,10]. A previous report by Kim *et al*. showed that muscle-derived Gr1 (dim) CD11b+ myeloid cells enhance neoangiogenesis in an ischaemic hindlimb mouse model [11]. In a recent report, it was demonstrated that intravenous administration of Gr-1+CD11b+ myeloid cells increased neoangiogenesis and improved heart function after heart infarction in mice [12]. Together, the results of these studies indicate that Gr-1+CD11b+ myeloid cells may serve as a cell source for neoangiogenesis, thus enhancing diabetic wound healing. Indeed, local administration of Gr-1+CD11b+ myeloid cells was reported to contribute to injury-induced neovascularization in diabetic mice [13]. In this study, a mouse model of dorsal window chamber, hindlimb ischaemia and ear-punch injury in diabetic or non-diabetic mice were used to further investigate whether Gr-1+CD11b+ myeloid cells efficiently home to the site of injury *via* intravenous administration and enhance diabetic wound healing by neoangiogenesis.

## Materials and methods

### Animals

C57BL/6 male mice, green fluorescent protein (GFP) mice (C57BL/6 background) and diabetic *db/db* mice (C57BL/6 background) were purchased from the Jackson Laboratory (Bar Harbor, ME, USA). All procedures involving animals were approved by Vanderbilt University Animal Care and Use Committee and the Liaoning Medical University Animal Care and Use Committee.

### Flowcytometry and single cell sorting

Single cell suspensions were made from spleens of non-diabetic mice. For Gr-1+CD11b+ myeloid cell sorting, a previously published protocol was followed [14]. These cells were labelled with fluorescence-conjugated antibodies (BD Pharmingen, San Diego, CA, USA) and isotype-matched IgG controls. The cells were sorted with a FACStarPlus flow cytometer (Becton Dickinson, Mountain View, CA, USA) and analysed on a FACScan flow cytometer (Becton Dickinson) with BD cell quest software.

### Animal experiments in non-diabetic mice

#### Dorsal window model for evaluation of neoangiogenesis by intravenous administration of Gr-1+CD11b+ myeloid cells

The *in vivo* vascular window model was performed as previously described, involving a metal frame applied to the back skin fold of non-diabetic mice [15]. Briefly, a 0.8-cm-diameter hole was dissected in one side of the epithelial surface of the dorsal skin flap. The underlying tissue was dissected away until a fascial plane with associated vasculature remained. A total number of 10^6^ Gr-1+CD11b+ myeloid cells in 100 μl of saline were injected into the tail vein after the window frame was installed in the mice. Control mice were injected with 100 μl saline only. The vasculature at the site of window chamber was examined thoroughly each day. At the tenth day, the vasculature was observed under light microscope and photographed.

#### Tracing of myeloid cells after intravenous injection

The Gr-1+CD11b+ myeloid cells were marked with the cell tracking dye, PKH-26 and injected into the tail vein after the window frame was installed in the non-diabetic mice. The homing of Gr-1+CD11b+ myeloid cells to the site of injury was observed under the fluorescent microscope (Olympus IX71, Tokyo, Japan) through the window chamber and photographed.

#### *In vivo* inhibition of CXCR4

The inhibition of CXCR4+ was investigated *in vivo* according to the method previously described by Abbott *et al*. [16]. Briefly, before intravenous delivery, Gr-1+CD11b+ myeloid cells marked with PKH-26 were incubated for 30 min. at 37°C with 5 μg/ml of a CXCR4 inhibitor named plerixafor (AMD3100). The cells were washed in PBS three times and then immediately delivered intravenously. After 24 hrs, the homing of Gr-1+CD11b+ myeloid cells to the site of the window chamber was observed under fluorescent microscope and photographed. The number of myeloid cells was calculated in five randomly selected fields and counted by an investigator blind to the study protocol.

### Animal experiments in diabetic mice

#### Tracing of myeloid cells after intravenous injection

The Gr-1+CD11b+ myeloid cells were sorted from spleens of GFP mice, and injected into the tail vein after the window frame was installed in the diabetic mice. After 24 hrs, the mice were injected intravenously with a solution of high molecular weight dextran tetramethylrhodamine (Molecular Probes, Eugene, OR, USA). Immediately after the injection of dextran, the homing of Gr-1+CD11b+ myeloid cells to the site of injury and vasculature was observed under the confocal microscope (Zeiss LSM 510, Munchen, Germany) through the window chamber and photographed.

#### Lower limb ischaemic model

Lower limb ischaemia in diabetic mice was created according to the method previously described [17]. Mice were anesthetized with intraperitoneal (i.p) injection of 160 mg/kg pentobarbital. To create the hindlimb ischaemia model, the femoral artery in the right lower limb was excised, from just above the deep femoral artery to the popliteal artery. For cell transplantation intervention, 10^6^ Gr-1+CD11b+ myeloid cells suspended in 100 μl of saline were injected into the tail vein after the surgical procedure.

#### Laser Doppler measurement of ischaemic limb

Blood flow recovery in the ischaemic limb in diabetic mice was serially evaluated with laser Doppler perfusion imaging over the course of 3 weeks post-operatively. Calculated blood perfusion (relative units) is expressed as the ischaemic (right)/normal (left) limb blood perfusion ratio [18,19].

#### Ear-punch injury

The ear-punch injury was performed according to the method described by Cho *et al*. [20]. Briefly, a hole of 3 mm in diameter was made by a punch in the centre of the ears of diabetic mice. A total number of 10^6^ Gr-1+CD11b+ myeloid cells in 100 μl of saline were injected into the tail vein. The size of the punched hole was measured and compared for three consecutive weeks. At the third week, the mice were injected intravenously with a solution of high molecular weight dextran tetramethylrhodamine (10,000 MW) and killed. The ears of the mice were dissected and photographed under a normal and a fluorescent microscope.

### Immunohistochemistry

Immunostaining was performed as previously described [21]. Briefly, cryosections of limb in diabetic mice after 3 weeks of ischaemia were fixed with 4% paraformaldehyde, permeabilized with 0.1% Triton X-100 in PBS, and blocked with 3% bovine serum albumin in PBS. The sections were incubated with rabbit monoclonal antibody to CD31 (Pharmingen, San Jose, CA, USA) at a concentration of 1:100 for 60 min., washed with PBS, and then incubated with goat anti-rabbit IgG secondary antibody at a concentration of 1:200. The Vector ABC kit (Vector Laboratories, Burlingame, CA, USA) was used according to the manufacturers' instruction and stained with DAB. Nuclei were counter-stained with haematoxylin. All images were captured with a light microscope. Capillary density was calculated in five randomly selected fields and counted by an investigator blind to the study protocol. Capillary density was expressed as number/muscle fibre.

### Western blot

Immunoblotting was performed with standard protocols as previously described [21]. The tissue from the chamber of dorsal window of diabetic and non-diabetic mice was harvested at 24 hrs after the model was established, placed into 4 ml lysis buffer, incubated for 5 min. on ice, and homogenized with a mixing homogenizer (Kinematica AG, Littau, Switzerland). The homogenates were heated at 95°C for 10 min. and centrifuged at 12,000 × g for 10 min. The protein concentration was measured by the BCA method (Pierce, Rockford, IL, USA). Aliquots of 40 μg of each sample were loaded on 15% SDS polyacrylamide gel, subjected to electrophoresis and transferred onto nitrocellulose membrane. The membranes were blocked with 5% non-fat milk in Tris buffer saline containing 0.1% Tween 20 at room temperature (RT) for 1 hr, and then probed with goat polyclonal antibody to SDF-1 (sc-6193; Santa, Cruz Biotechnology, Santa Cruz, CA, USA) at a concentration of 1:500 at 4°C overnight, followed by incubation with horseradish peroxidase–conjugated anti-goat IgG (Zymed, South San Francisco, CA, USA) at a concentration of 1:1000 at RT for 1 hr. The horseradish peroxidase was detected with a chemoluminescence ECL-Plus kit (Amersham Biosciences UK, Little Chalfont, UK). Tubulin was detected as an internal protein loading control.

### Statistical analysis

Data are expressed as mean ± SD. Statistical comparisons were performed with anova followed by Bonferroni/Dunn testing. A *P* < 0.05 was considered statistically significant.

## Results

### Gr-1+CD11b+ myeloid cells efficiently home to the site of injury and increase neoangiogenesis after intravenous injection in non-diabetic mice

**Figure 1:**
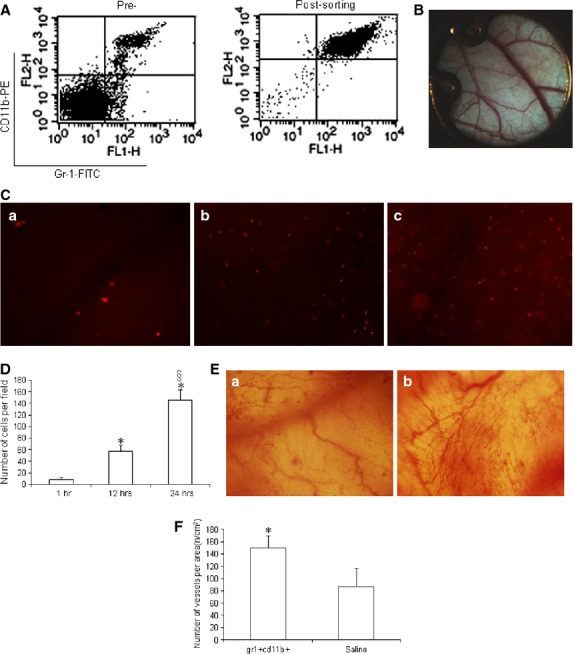
Gr-1+CD11b+ myeloid cells homed to the site of injury after intravenous administration and increased neoangiogenesis at the site of injury in non-diabetic mice. (A) flow cytometry analysis of Gr-1+CD11b+ myeloid cells pre- and post-sorting, which showed the purity of the Gr-1+CD11b+ myeloid cells is ∽95%; (B) establishment of the dorsal window chamber in mice; (C) Gr-1+CD11b+ myeloid cells homed to the site of window chamber injury after intravenous tail vein injection. (a) Gr-1+CD11b+ myeloid cells homed to the site of window chamber 1 hr after injection; (b) Gr-1+CD11b+ myeloid cells homed to the site of window chamber 12 hrs after tail vein injection; (c) Gr-1+CD11b+ myeloid cells homed to the site of window chamber 24 hrs after tail vein injection. Magnification ×40 for each image. (D) With the increasing of time, the number of Gr-1+CD11b+ myeloid cells homing to the site of injury significantly increased. (E) Neoangiogenesis at the window chamber after intravenous injection of Gr-1+CD11b+ myeloid cells. (a) saline treatment; (b) Gr-1+CD11b+ myeloid cells treatment. Magnification ×40 for each image. (F) Gr-1+CD11b+ myeloid cells significantly increased neoangiogenesis at the site of injury, *P < 0.01 *versus* 1 hr; ^§^*P* < 0.01 *versus* 1 and 12 hrs, *n* = 5 in each group.

The Gr-1+CD11b+ myeloid cells were sorted from the spleens of non-diabetic mice. The purity of Gr-1+CD11b+ myeloid cells after sorting was ∽95% (Fig.1A). To investigate whether Gr-1+CD11b+ myeloid cells increase neoangiogenesis at the site of injury, the dorsal window model in non-diabetic mice was established (Fig.1B). The Gr-1+CD11b+ myeloid cells were injected into tail vein of mice to determine if injected Gr-1+CD11b+ myeloid cells efficiently home to the window chamber site of injury, and the homing of Gr-1+CD11b+ myeloid cells to the site of injury was monitored in a real-time manner. By tracing Gr-1+CD11b+ myeloid cells marked with PKH-26 for 24 hrs, Gr-1+CD11b+ myeloid cells were found to efficiently home to the site of the window chamber after intravenous injection (Fig.1C and D). Furthermore, 10 days after the intravenous injection of Gr-1+CD11b+ myeloid cells, blood vessel branches at the site of the window chamber significantly increased, compared with the saline-treated group, suggesting that the Gr-1+CD11b+ myeloid cells increase neoangiogenesis at the site of injury (Fig.1E and F).

### Gr-1+CD11b+ myeloid cells home to the site of injury *via* the SDF-1/CXCR4 axis

**Figure 2:**
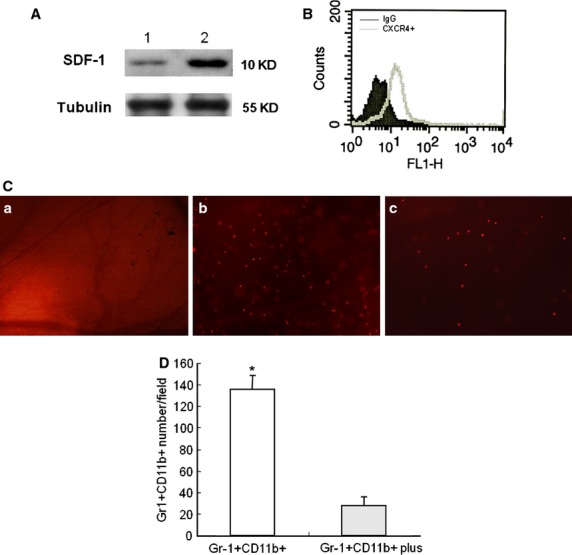
The CXCR4/SDF-1 axis plays an important role in the homing of Gr-1+CD11b+ myeloid cells to the site of injury. (A) Representative Western blot detection of SDF-1 in the tissue at the site of injury 24 hrs after establishment of the window chamber model. Lane 1, normal skin tissue; lane 2, skin tissue at the site of the window chamber injury. Experiments were repeated three times. (B) Flowcytometry measurement of CXCR4 expression in Gr-1+CD11b+ myeloid cells; (C) tracing of the homing of Gr-1+CD11b+ myeloid cells to the site of window chamber injury. (a) saline control; (b) Gr-1+CD11b+ myeloid cells with no treatment; (c) Gr-1+CD11b+ myeloid cells treated with the CXCR4 inhibitor, AM3100. Magnification ×40 for all images. (D) Inhibition of CXCR4 significantly decreased the homing of Gr-1+CD11b+ myeloid cells to the site of injury *P < 0.01.

To investigate the specific targeting of Gr-1+CD11b+ myeloid cell to the site of injury, SDF-1 expression in tissue at the site of injury was investigated by Western blot 24 hrs after establishment of the window chamber model in non-diabetic mice. The expression of CXCR4 in Gr-1+CD11b+ myeloid cells was investigated by flowcytometry. The SDF-1 increased in the tissue at the site of window chamber (Fig.2A). There was robust CXCR4 expression in the Gr-1+CD11b+ myeloid cells (Fig.2B). This indicates that the SDF-1/CXCR4 axis may play a role in the homing of Gr-1+CD11b+ myeloid cells to the site of injury. To further evaluate the role of SDF-1/CXCR4 axis in the homing of Gr-1+CD11b+ myeloid cells to the site of injury, the expression of the CXCR4 receptor was blocked by AMD3100 and the homing of Gr-1+CD11b+ myeloid cells to the site of window chamber traced. The plerixafor, AMD3100, significantly inhibited the homing of Gr-1+CD11b+ myeloid cells to the site of injury of the window chamber (Fig.2C and D). This indicates that the SDF-1/CXCR4 axis plays an important role in the homing of Gr-1+CD11b+ myeloid cells to the site of injury.

### SDF-1 expression is down-regulated at the site of injury in diabetic mice, but increases compared with its basal level

**Figure 3:**
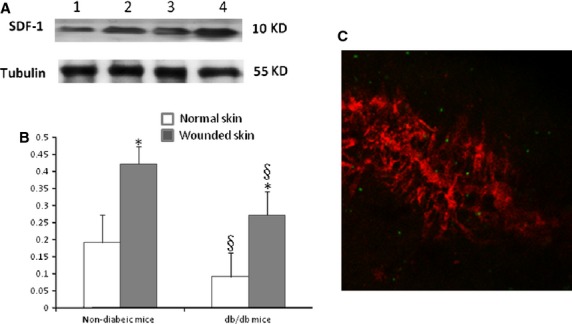
SDF-1 expression at the diabetic wound site was down-regulated compared with non-diabetic mice; however, SDF-1 expression was increased compared with its basal level. (A) Western blot detection of SDF-1 expression at the site of injury. Lane 1, normal skin tissue of diabetic mice; lane 2, skin tissue of diabetic mice at the site of the window chamber; lane 3, normal skin tissue of non-diabetic mice mice; lane 4, skin tissue of non-diabetic mice at the site of the window chamber. The experiments were repeated three times. (B) Densitomery of SDF-1 expression at the injury site 24 hrs after the window chamber model was established; *P < 0.01 *versus* normal skin; ^§^*P* < 0.05 *versus* non-diabetic mice, *n* = 5 in each group. (C) Gr-1+CD11b+ myeloid cells home to the site of injury after intravenous administration. Red colour indicates the microvessels injected with dextran tetramethylrhodamine imaged with confocal microscope at the site of injury, green colour indicates the Gr-1+CD11b+ myeloid cells with GFP homing to the site of injury; magnification ×40.

To investigate whether diabetic mellitus affects expression of SDF-1 at the site of injury, Western blot was performed to detect the expression of SDF-1 at the site of window chamber in diabetic mice. The results of this study show that whilst expression of SDF-1 in the diabetic wound was down-regulated compared with the non-diabetic mice, the expression of SDF-1 increased compared with its basal level (Fig.3A and B). To provide evidence that Gr-1+CD11b+ myeloid cells home to the site of injury after intravenous administration in diabetic mice, the Gr-1+CD11b+ myeloid cells were sorted from spleens of GFP mice and injected into the tail vein of diabetic mice. The results clearly showed that green fluorescent Gr-1+CD11b+ myeloid cells homed to the window chamber (Fig.3C).

### Gr-1+CD11b+ myeloid cells improve blood flow recovery and capillary density of ischaemic limb in diabetic mice

**Figure 4:**
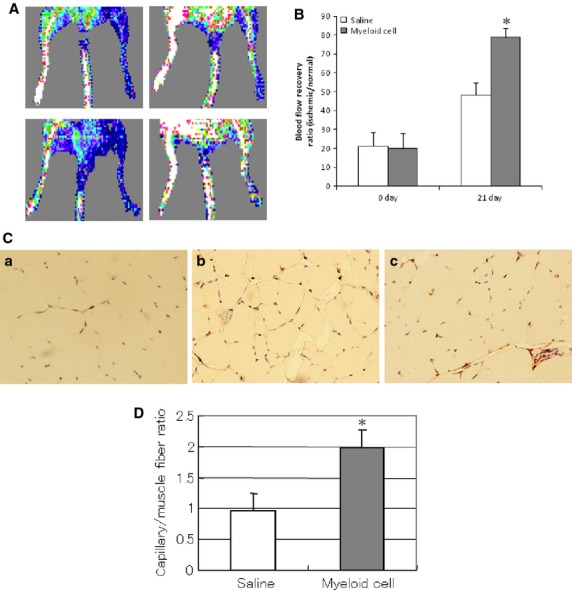
Intravenous injection of Gr-1+CD11b+ myeloid cells improved blood flow recovery and capillary density in the ischaemic limb of diabetic mice. (A) Representative laser Doppler imaging of blood flow recovery of ischaemic limb; left panel, ischaemic limb immediately after hindlimb ischaemia; right panel, ischaemic limb after 21 days of hindlimb ischaemia; upper panel, ischaemic limb from mice intravenously injected with saline; lower panel, ischaemic limb from mice intravenously injected with Gr-1+CD11b+ myeloid cells. (B) Gr-1+CD11b+ myeloid cells significantly improved blood flow recovery of the ischaemic limb compared with the saline control, *P < 0.01, *n* = 5 in each group. (C) Representative photographs of immunostaining of CD31 in the ischaemic limb muscle. (a) isotype control of ischaemic limb of mice; (b) ischaemic limb from mice intravenously injected with saline; (c) ischaemic limb from mice intravenously injected with Gr-1+CD11b+ myeloid cells. Magnification ×200 for each image. (D) Gr-1+CD11b+ myeloid cells significantly increased capillary density of the ischaemic limb, *P < 0.01 *versus* saline, *n* = 5 in each group.

As diabetic foot ulcer is related to lower limb ischaemia, the Gr-1+CD11b+ myeloid cells were injected into the tail vein to investigate whether the cells can improve blood recovery in the ischaemic limb of diabetic mice. Blood flow recovery was determined by serial laser Doppler measurement. The Gr-1+CD11b+ myeloid cells significantly improve the blood flow recovery in the ischaemic limb (78.9 ± 4.9% *versus* 48.3 ± 6.4% control, at 3 weeks, *P* < 0.01, *n* = 5 in each group, Fig.4A and B). The immunostaining of CD31 showed that Gr-1+CD11b+ myeloid cells significantly increase capillary density in the ischaemic limb of diabetic mice (Fig.4C and D).

### Gr-1+CD11b+ myeloid cells promote wound healing by increasing neoangiogenesis in diabetic mice

**Figure 5:**
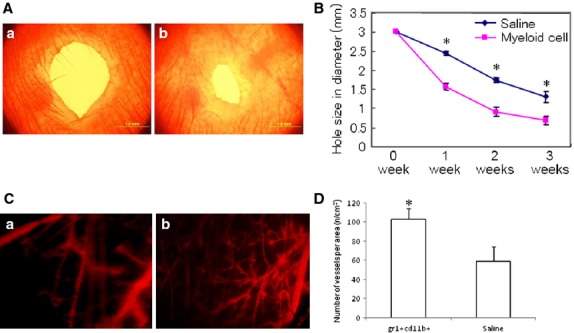
Intravenous injection of Gr-1+CD11b+ myeloid cells enhanced wound healing in diabetic mice. (A) Representative wound size of ear 3 weeks after ear-punch injury. (a) wounded ear from mice intravenously injected with saline; (b) wounded ear from mice intravenously injected with Gr-1+CD11b+ myeloid cells; scale bar = 1 mm. (B) Time course of ear wound healing. Gr-1+CD11b+ myeloid cells significantly enhanced wound healing compared with saline control, *P < 0.01, *n* = 5 in each group. (C) Representative imaging of neoangiogenesis in the ear 3 weeks after ear-punch injury (red colour indicated the blood vessels filled with dextran tetramethylrhodamine). (a) wounded ear from mice intravenously injected with saline; (b) wounded ear from mice intravenously injected with Gr-1+CD11b+ myeloid cells. Magnification ×40 for each image. (D) Gr-1+CD11b+ myeloid cells significantly increased neoangiogenesis in the wound ear. *P < 0.01 *versus* saline, *n* = 5 in each group.

Ear-punch injury in mice was used as the model to investigate the wound healing process. Intravenous injection of Gr-1+CD11b+ myeloid cells significantly decreased the size of the punched hole in the ear of mice (1.57 ± 0.08 mm *versus* 2.44 ± 0.05 mm control at 1 week, *P* < 0.01; 0.69 ± 0.11 mm *versus* 1.30 ± 0.14 mm control at 3 weeks, *P* < 0.01, *n* = 5 in each group, Fig.5A and B). This indicates that Gr-1+CD11b+ myeloid cells promote the diabetic wound healing. Fluorescent imaging showed that Gr-1+CD11b+ myeloid cells significantly increased neoangiogenesis in the wounded ear of diabetic mice (Fig.5C and D).

## Discussion

In this study, Gr-1+CD11b+ myeloid cells efficiently homed to the site of injury after intravenous administration and increased neoangiogenesis at the wound site. The Gr-1+CD11b+ myeloid cells targeted the site of injury by the mutual effect of SDF-1 and CXCR4. Furthermore, intravenous administration of Gr-1+CD11b+ myeloid cells improved blood flow recovery of the ischaemic limb and enhanced wound healing by neoangiogenes in diabetic mice. This provides significant evidence that intravenous administration of Gr-1+CD11b+ myeloid cells could be used as an alternative and practical method to enhance diabetic wound healing.

Several myeloid cell populations including monocytes, macrophages and neutrophils play important roles in neovascularization in ischaemic tissues [22–24]. It has been reported that common myeloid progenitors (CMPs), which arise from pluripotent haematopoietic stem cells, give rise to endothelial cells [25]. Wara *et al*. reported that CMPs preferentially differentiate into pro-angiogenic cells compared to megakaryocyte–erythrocyte progenitors and common lymphoid progenitors [26]. Gr-1+CD11b+ myeloid cells are heterogeneous and include a spectrum of myeloid lineage cells from immature cells to terminally differentiated neutrophils [27,28]. Thus, Gr-1+CD11b+ myeloid cell may be a potential cell source for neoangiogenesis. Indeed, we previously reported that Gr-1+CD11b+ myeloid cells promote neoangiogenesis in tumour [27] as well as in infarct heart [12]. Recently, Kim *et al*. demonstrated that Muscle-derived Gr1(dim)CD11b(+) myeloid cells enhance neovascularization in an ischaemic hindlimb mouse model [11]. In this study, we further showed that Gr-1+CD11b+ myeloid cells derived from the spleen of normal mice improve blood flow recovery of hind ischaemic limb and enhance wound healing *via* increasing neoangiogenesis in diabetic mice. Although the mechanisms of neoangiogenesis by Gr-1+CD11b+ myeloid cells are not fully elucidated, in a previous report, we demonstrated that Gr-1+CD11b+ myeloid cells increase angiogenesis by releasing matrix metalloproteinase-9 [27]. In a report by Grunewald *et al*., they also found that myeloid cells facilitate angiogenesis by releasing matrix metalloproteinase-9 [9]. Moreover, some of the Gr-1+CD11b+ myeloid cells were found to incorporate into the vascularture, which means that Gr-1+CD11b+ myeloid cells also increase neoangiogenesis by vasculogenesis [12,27].

The homing of cells to the site of injury is one of the important events in bone marrow–derived cell therapy to wound healing. Some cytokines such as VEGF promote mobilization and homing of progenitor cells to hypoxic site [29,30]. Recently, chemokines are also found to be important factors for this process. Ceradini *et al*. demonstrated that recruitment of CXCR4-positive cells to regenerating tissues is mediated by hypoxic gradients *via* HIF-induced expression of SDF-1[31]. Grunewald *et al*. revealed that VEGF mobilizes bone marrow derived CxCR4+ cells and induces SDF-1 expression in perivascular fibroblast cells in the target organ. SDF-1 retains incoming CXCR4+ cells, which produce pro-angiogenic activities that stimulate proliferation of resident endothelium [9]. In this study, SDF-1 increased at the site of injury in non-diabetic mice. A similar result was implicated in the ischaemic hindlimb in non-diabetic mice [32]. In addition, it was found that CXCR4 is robustly expressed in Gr-1+CD11b+ myeloid cells and inhibition of CXCR4 by AMD3100 decreased the homing of Gr-1+CD11b+ myeloid cells to the site of injury. These results indicate that the SDF-1/CXCR4 axis plays an important role in the homing of Gr-1+CD11b+ myeloid cells to the site of injury after intravenous administration. In diabetic mice, the expression of SDF-1 was down-regulated at the wound site compared with normal mice; however, the expression of SDF-1 was found to increase at the diabetic wound site compared with its basal level. This is consistent with results of a previous study in a middle cerebral artery occlusion model in *db/db* mice by Chen *et al*. [33]. Chen *et al*. found that transfusion of CXCR4-primed endothelial progenitor cells reduces cerebral ischaemic damage and promotes repair in *db/db* mice even though the SDF-1 expression at the ischaemic site was down-regulated [33]. In this study, intravenous administration of Gr-1+CD11b+ myeloid cells increased neoangiogenesis and enhanced wound healing in the diabetic *db/db* mice. The primary reason for this may be the robust expression of CXCR4 in Gr-1+CD11b+ myeloid cells that may compensate for the effect of down-regulation of SDF-1 expression in the diabetic wound.

Over the last decade, successful pre-clinical studies have created great excitement about the potential use of MSCs for patients with diabetic wounds [34]. However, before translation from the bench to clinical usage of MSCs, several barriers need to be overcome. One barrier to MSC therapy for wound healing is the method of delivering the cells. Systemic delivery is an attractive option; however, the homing and engrafting efficiency of MSCs to the site of injury is not satisfactory [35]. When adopting the method of local administration, improving the viability and efficiency of MSCs migration into the wound bed is always an obstacle that needs to be overcome [36]. Thus, a stem cell population that can efficiently home and engraft to the site of injury appears to be an ideal selection. In this study, using a murine dorsal window model, the homing of Gr-1+CD11b+ myeloid cells to the site of injury was monitored in a real-time manner after intravenous injection. The Gr-1+CD11b+ myeloid cells efficiently homed to the site of injury and promoted neoangiogenesis after intravenous injection. This characteristic of Gr-1+CD11b+ myeloid cells makes these cells a convenient and efficient method of cell delivery in clinical practice.

Gr-1+CD11b+ cells contribute to injury-induced neovascularization. However, diabetic-derived Gr-1+CD11b+ cells fail to stimulate neovascularization *in vivo* and have aberrant proliferative, chemotaxis, adhesion and differentiation potential. Sustained expression of Hoxa3 in diabetic-derived Gr-1+CD11b+ cells reverses their diabetic phenotype [13]. Consequently, before attempting to determine an effective and safe way to manipulate the diabetic-derived Gr-1+CD11b+ cells, thus reversing their diabetic phenotype, the usage of Gr-1+CD11b+ cells from healthy donors is one method that could be chosen to enhance diabetic wound healing in clinical practice. Further studies are required to evaluate the feasibility and safety of using non-diabetic Gr-1+CD11b+ cells to enhance wound healing in diabetic patients.

In conclusion, Gr-1+CD11b+ myeloid cells efficiently home to the site of injury after intravenous administration and improve diabetic wound healing by increasing neoangiogenesis. The SDF-1/CXCR4 axis plays an important role in the homing of the Gr-1+CD11b+ myeloid cells to the site of injury. The Gr-1+CD11b+ myeloid cells could be used as a potential cell source in the enhancement of diabetic wound healing.
